# mHealth for Anemia Reduction: Protocol for an Entertainment Education–Based Dual Intervention

**DOI:** 10.2196/26252

**Published:** 2021-11-22

**Authors:** Ichhya Pant, Rajiv Rimal, Hagere Yilma, Jeffrey Bingenheimer, Erica Sedlander, Sibabrata Behera

**Affiliations:** 1 Department of Prevention and Community Health School of Public Health George Washington University Washington, DC United States; 2 Department of Health, Behavior and Society Bloomberg School of Public Health Johns Hopkins University Baltimore, MD United States; 3 DCOR Inc Bhubaneshwar, Odisha India

**Keywords:** mHealth, interactive, voice response, entertainment, education, rural, anemia, bystander, violence against women

## Abstract

**Background:**

More than half of the women of reproductive age (aged 15-49 years) are anemic in India. The uptake of and adherence to iron folic acid (IFA) supplements remain low despite sustained efforts to increase their use. With India’s burgeoning digital environment, mobile phones offer a potential medium for increasing their uptake, especially when combined with interactive voice messages that deliver entertaining stories infused with norms-based educational messages.

**Objective:**

This study aims to investigate whether a norms-based entertainment education mobile health intervention can increase self-efficacy for IFA adherence among women of reproductive age in Odisha, India.

**Methods:**

Mobile reduction in anemia through normative innovations (mRANI) is a randomized 2-arm study that includes assessments before and after the intervention. All study participants will be recruited from the intervention arm of the parent reduction in anemia through normative innovations trial only. Although the usual practice is to randomize participants either to a treatment arm or a usual care control arm, we will assign the mRANI control group to another entertainment education–based treatment group that is designed to improve bystander intervention to reduce violence against women. Data collection for the mRANI study is embedded in the parent trial and will include baseline and end line assessments. The primary outcomes are self-efficacy for IFA adherence and violence against women–related bystander intervention. The inclusion criteria for the mRANI study are participation in the parent trial and phone ownership. Women (approximately n=400) who meet the mRANI inclusion criteria will be randomly assigned to the IFA arm or the bystander arm. Ordinary least squares regression with robust SEs will be conducted to assess between-group comparisons at the end line. A mediation analysis will be conducted to examine whether social norms and interactivity mediate the relationship between intervention exposure and primary outcomes in both arms. Real-time monitoring data will offer insights into intervention receptivity and audience engagement.

**Results:**

Data collection for the mRANI study is integrated within the parent trial. Household surveys were conducted between February and March of 2021. Responses on the mRANI study’s primary and secondary outcomes were collected from 381 participants. The data analysis is expected to be completed by October 2021.

**Conclusions:**

This study will provide evidence on whether a mobile health norms–based entertainment education intervention can increase self-efficacy for IFA adherence and violence against women–related bystander intervention.

**International Registered Report Identifier (IRRID):**

PRR1-10.2196/26252

## Introduction

### Endemic Anemia Among Women of Reproductive Age

More than half of the women of reproductive age (aged 15-49 years) in Odisha, India, are anemic [1]. Low dietary iron intake, reduced iron absorption, and iron loss contribute to iron deficiency among women of reproductive age [2]. Anemia diminishes work productivity [3,4], increases the risk of adverse birth outcomes [5], and hinders the healthy development of children of pregnant mothers with anemia [6]. It is often not explicitly identified by women, but they do report high levels of fatigue [3]. The reduction in anemia through normative innovations (RANI) project [2] investigates the potential of an adaptive, multilevel social norms–based behavior change intervention to reduce anemia among women of reproductive age in Odisha.

The RANI project targets nonpregnant women who are currently underserved by the government’s anemia reduction programs. Various government initiatives in India (eg, National Nutritional Anemia Control Program, National Iron Plus Initiative, and Anemia Mukt Bharat) provide iron syrup to infants and iron tablets to both adolescent girls in schools and pregnant women linked with the health system. Non–school-going women of reproductive age and those not pregnant are neglected in most national and state programs, although they are listed as government priority populations. Research on this group is also scant: for example, the Indian National Family Health Survey collects data only for pregnant women [1,7]. Adherence to the recommended iron folic acid (IFA) regimen is low even among pregnant women specifically targeted by various government programs. The 2015-2016 National Family Health Survey found that 91% of mothers reported receiving IFA supplements; however, only 30.3% reported consuming them for ≥100 days during pregnancy [1].

Considering these insights, the RANI project has adopted a social norms–based approach for improving IFA uptake and adherence. The strategy includes the following: (1) participatory learning delivered through in-person activities and games; (2) a series of tablet- and computer-based health communication videos that target various audiences (pregnant women, nonpregnant women, husbands, and mothers-in-law), followed by discussion sessions; and (3) monthly community-based testing of hemoglobin levels of 15 women in each village, followed by a discussion about trends in anemia and village-level comparisons (based on the hemoglobin readings) with neighboring communities. A full protocol of the intervention and evaluation design is published elsewhere [2], as is the process used to develop the intervention from formative evaluation findings (Table 1) [7].

**Table 1:** Intervention components, exposure, and sample size.

Group	Exposure	Components	Sample size, n
RANI^a^ control	Usual care	None (usual care)	2000
RANI treatment, not in mRANI^b^	All RANI treatment components	Interactive learning sessions, RANI communication videos, and hemoglobin testing	1700
RANI treatment+mRANI treatment (mIFA^c^)	All RANI treatment components+entertainment education and mRANI IFA^d^ adherence arm	All components in RANI treatment that are not in mRANI+entertainment education story and mHealth^e^ anemia intervention	206
RANI treatment+mRANI control (mBI^f^)	All RANI treatment components+entertainment education and mRANI bystander intervention arm	All components in RANI treatment, not in mRANI+entertainment education story and mHealth bystander intervention	205

^a^RANI: reduction in anemia through normative innovations.

^b^mRANI: mobile reduction in anemia through normative innovations.

^c^mIFA: mobile iron folic acid.

^d^IFA: iron folic acid.

^e^mHealth: mobile health.

^f^mBI: mobile bystander intervention.

### Emergence of a Dual Burden of Anemia and Violence Against Women

In the formative evaluation phase, the RANI project sought to identify key issues faced by women in Odisha that would shed light on their anemia-specific behaviors. A primary theme that emerged was the role of gender norms, which illustrated the multiple ways through which women experienced social, structural, and individual-level disadvantages and barriers [7]. Unprompted by the research team, a key issue that respondents brought up was the high level of violence against women in their communities.

Violence against women is a global problem, with more than 1 in 3 women worldwide reporting physical or sexual violence by an intimate partner or nonpartner [8-11]. This estimate precludes violence against women that manifests in other forms, such as gender-based household or familial violence and maltreatment and emotional or economic domestic violence [11-14]. Lived experiences of multiple forms of violence toward women are especially egregious in India, where approximately 1 out of 2 women report having survived physical violence, experienced psychological abuse, or experienced multiple forms of violence [11-15]. Similarly, in rural Odisha, 2 out of 5 women have reported various forms of violence directed toward them [16]. Self-disclosure of violence to a friend or relative is far more commonplace (40%) than formal sources of support (7%) [13,17]. Most occurrences of violence against women continue to be undisclosed because of stigma and fear of reprisals [13,17]. Because of lax enforcement of extant laws, social and gender norms appear to condone widespread violence toward women [13,17-21]. As such, interventions that focus on societal and collective transformation (eg, bystander interventions) by influencing inequitable social and gender norms to reduce violence against women are necessary [13,17-21]. Although the RANI project was not explicitly funded to address this issue, as we note in the section below, we have found a creative way to incorporate it into our intervention design.

### Mobile RANI: Bridging the Gap With Mobile Health Infused With Entertainment Education

Recent years have seen tremendous growth in both the scope and number of mobile health (mHealth) interventions worldwide, especially in low-resource settings [22]. Many mHealth programs have demonstrated potential for improving a variety of health outcomes related to sexual and reproductive health, maternal and child health, and medication adherence in urban and rural settings in several countries in Asia [23-25] and Africa [26]. However, a dearth of high-quality trials in low-resource settings limits evidence of their efficacy, which is a key barrier for institutional adoption and uptake of digital health solutions [22,27].

India is one of the largest and fastest growing markets for information and communication technologies, with 1.2 billion phone and internet subscriptions, respectively [28]. To the best of our knowledge, only 2 prior mHealth interventions have been conducted to promote IFA adherence in India. They also focused only on pregnant women with anemia, and thus, the vast majority of women of reproductive age remained neglected [29]. One of these efforts used personalized automated voice calls from health care providers [29], whereas the other relied on live calls [30]. Both studies not only demonstrated the potential of using voice calls as a medium for promoting IFA adherence but also highlighted a number of inherent challenges. The most critical challenge faced by Pai et al [29] was the inability to retain program participants, which led to nonsignificant changes in IFA adherence despite a positive trend. To maximize retention, they recommended increasing participant involvement in addition to appealing to the listener’s self-efficacy or perceived ability to adhere to IFA. Studies in the field of behavior change have also shown the important role that self-efficacy can play in predicting behaviors; people are most likely to engage in a behavior when they feel confident that they can do so in the face of barriers [31].

Following these recommendations, the mobile RANI (mRANI) study will center its efforts on improving self-efficacy for IFA adherence as one of its primary goals. We have powered our mRANI study for this outcome as well. As described below, the control arm for the mRANI study is another intervention that focuses on reducing violence against women by promoting bystander intervention, which also uses self-efficacy as its outcome.

### Conceptual Background

Our overall approach is anchored in the entertainment education (EE) literature [32,33]. The idea behind an EE approach is that when people are being entertained by media, their defenses against persuasive attempts are significantly lower (than they would be while viewing an appeal explicitly trying to change their attitude or behavior). This provides interventions with an entrée for embedding health-related information in the main storyline. EE is rooted in an oral and performing arts tradition, and its research and application have evolved in parallel with the ongoing movement for digital innovation and social equity and change [32,33]. It leverages the power of narratives and storytelling to exact a transformative effect on its audiences [34]. Interventions are most commonly delivered via soap operas on television or radio, commercial music, and music videos. Comic books, films, news media, radio talk shows, mobile vans, and telephone hotlines also serve as supplementary channels [33].

As the research and practice of EE has evolved, so has the theoretical scholarship focused on understanding how it works [34]. Scholars are expanding beyond individual factors, such as the direct effects of role modeling or observational learning, and turning their attention to the influence of social norms and social consequences on EE effects [32,34-38]. On the basis of empirical evidence, many such programs have successfully transformed social norms related to tissue and organ donation in Korea [34,35], gender norms related to sexual and reproductive health in 6 states within India [36], and stigma related to HIV/AIDS [35-38]. Building on the efficacy of these prior studies, the mRANI study will run an EE mHealth program delivered exclusively via mobile phones to shift social norms related to IFA consumption and promote self-efficacy for IFA adherence.

Another component of the intervention we explore in this study is the role of interactivity in EE. We propose two competing mechanisms of change that we will test in this project. On the one hand, when the program promotes interactivity, user engagement with the content is expected to increase [39], with greater educational impact. On the other hand, interactivity can serve as a distraction from full immersion in story content, particularly when users are being entertained; this effect is more pronounced among those whose cognitive capacity has been compromised [40], as might be the case for people with anemia [41].

### Objective and Hypotheses

The objective of this study is to investigate the ability of a norms-based EE mHealth intervention in increasing self-efficacy for IFA adherence among women of reproductive age in Odisha, India. Our hypothesis is that women in the intervention arm will display greater positive improvements in self-efficacy, pre- to postintervention, than women in the control arm.

In addition to testing this hypothesis, we will also ask questions about the underlying process of change. In particular, our secondary aims are two-fold: to determine (1) the extent to which social norms and (2) interactivity mediate the relationship between intervention exposure and study outcomes.

## Methods

### Reciprocal Control Double Intervention Study Design

The mRANI study is a randomized 2-arm study that includes assessments before and after the intervention. All study participants are recruited only from the intervention arm of the parent RANI trial (the trial itself has a usual care control group and a social norms–based treatment group, see the study by Yilma et al [2] for the study protocol). Hence, we will randomize a subset of women in the parent intervention arm to either the treatment or control arm of the mRANI study. This design results in the following four groups: (1) the usual care control group (from the parent study arm) in which no RANI activities will occur, (2) the RANI intervention group not involved in the mRANI trial, (3) the RANI intervention group in the mRANI control arm, and (4) the RANI intervention group in the mRANI treatment arm. This allocation and each group’s exposure to the intervention components are shown in Table 1.

In this study, we introduce an innovative approach. Although the usual practice is to randomize participants either to a treatment arm or to a usual care control arm (this is also the design of the parent RANI trial), we will assign the mRANI control group to another EE-based treatment group designed to improve bystander intervention to reduce violence against women. We will call this the reciprocal control double treatment (RCDT) design. In this RCDT design, the treatment group for one intervention (mobile iron and folic adherence [mIFA] to improve IFA consumption self-efficacy) will serve as the control group for the other intervention (mobile bystander intervention [mBI] to improve bystander intervention self-efficacy), and vice versa.

The decision to center the second arm on bystander intervention (as opposed to another health issue) was based on two considerations. First, anemia and violence against women are empirically unrelated [42], which provides independence between the 2 arms. Second, violence against women is a significant public health issue in Odisha [16]—an issue that came up spontaneously in our formative assessment. Hence, incorporating this issue as an alternative intervention arm in our study seemed appropriate.

Taking this RCDT design into account, the hypothesis we proposed earlier will be tested by comparing changes from baseline to end line in self-efficacy to take IFA tablets in the treatment arm with the corresponding change in bystander self-efficacy to intervene in the control arm (and vice versa).

### Study Setting

The RANI project is currently underway in 2 blocks (administrative units below the district) in the Angul district of Odisha, India [2]. Most of our residents (83%) live in rural areas and are Hindu (94%) [43]. Anemia prevalence is higher among women with less education and those who belong to scheduled tribes [1]. Approximately two-thirds of women in Odisha are literate [43]. Anemia prevalence (50%) for Angul is similar to the state of Odisha and the overall countrywide anemia prevalence [1]. Likewise, according to recent estimates from a systematic review on violence against women in India, 30% of women reported having experienced multiple forms of abuse within the past year [11-15].

### Development and Implementation of mRANI

#### Overview

The mRANI intervention was developed based on the literature as well as quantitative analyses conducted using the RANI study’s formative and baseline surveys. Using these data, we developed a mobile ownership and user profile and identified an optimal intervention delivery channel. We first conducted a feasibility analysis to determine whether a phone-based intervention would be viable in our study sites. Our data from baseline assessments (n=1874) of the parent RANI trial revealed that 43.97% (824/1874) of women owned mobile phones, and 42.63% (799/1874) of those who did not outright own a mobile phone could borrow one from a close family member with permission. Only 13.39% (250/1874) of our study participants were classified as those without access to a mobile phone. Accounting for these structural limitations, to be eligible for the mRANI study, participants have to be mobile phone owners with an active phone line.

We also conducted formative research in the form of qualitative story testing to select an overall storyline for both arms. Once the overall storyline was finalized, we developed subplots for each arm, integrated them into the broader storyline, and translated them into scripts. Finally, we conceptualized and developed interactive components. Audio recording and production of both EE) programs took place as a final step. Multimedia Appendices 1 and 2 detail the methods for story testing and the overall mRANI EE narrative.

#### EE Program Delivery

EE programs will be delivered with 13 episodes over 5 weeks, each lasting approximately 3-5 minutes. A total of 3 new episodes will be released every week. The exact day and time of delivery will be personalized according to participant preferences. Episodes will be delivered using an interactive voice response (IVR) system that can provide individualized messages to participants and interact with them through voice recognition or a touch-tone keypad. Prior studies have demonstrated the effectiveness of the IVR system in mHealth interventions, especially in rural settings with populations with limited educational and digital literacy [29,44,45]. Calls received by participants will be free of cost.

#### Story Manipulations

The overall story was kept similar across both mRANI arms—that is, the mIFA arm and the mBI arm. However, we inserted messages about either taking IFA tablets (in the mIFA arm) or intervening to prevent violence as a bystander (mBI arm) at strategically chosen points in the storyline. Multimedia Appendix 3 includes episode summaries for both arms that convey how the broader storyline remained the same for the 2 arms whereas the subplots purposefully deviated to integrate educational and theoretical concepts.

#### Interactive Components

Interactive components for the mRANI study have been conceptualized at 3 levels: program-driven, audience-driven, and responsive interactions (Textbox 1). Program-driven interactions are initiated by the mRANI team and take place both before and during the intervention delivery. Examples of program-driven orientations include program jingles, episode teasers, and recaps. Audience-driven interactions are functionally built into the IVR system, allowing mRANI listeners to like and replay individual episodes. Finally, responsive interactions played at the end of each episode are also functionally built into the IVR system, allowing mRANI listeners to respond to prerecorded quizzes and questions on related prosocial themes, character identification, engagement and satisfaction with the narrative, and agreement with major plot points.

**Mobile reduction in anemia through normative innovations interactive components.**

**Program-driven interactions**

- Entertainment education (EE) program release announcements
- EE program jingles
- EE program episode teasers
- EE program episode recaps

**Audience-driven interactions**

- Like or replay EE episodes on an interactive voice response system
- Call a mobile reduction in anemia through normative innovations hotline

**Responsive interactions**

- Quizzes and questions on the following:
- Topics related to prosocial themes
- Character identification
- Engagement with the narrative
- Agreement with major plot points
- Satisfaction with the narrative
- Perception of the most important problems in the village

### Sample Size

We calculated the required sample size based on the findings of Constantino et al [46] in which they observed an improvement in self-efficacy in the treatment group (mean 3.30, SD 0.68) that was higher than that in the control group (mean 2.89, SD 0.6; *P*<.001). On the basis of similar assumptions, with a power of 0.8 and α of .05, our required sample size was 39 participants in each arm, which was rounded up to 40. To account for the subanalyses that we will conduct based on participants’ caste (tribal, scheduled caste, and other), we increased the sample size to 40×3=120 participants in each arm, for a total of 240 participants.

### Inclusion Criteria

All women of reproductive age who meet the inclusion criteria for the larger RANI trial will be eligible to participate in the mRANI trial. For the parent RANI trial, inclusion criteria included being a woman aged between 15 and 49 years, speaking Odiya, and residing in our study sites. In addition, for the mRANI trial subsample, we require participants to be mobile phone owners with an active phone line. Before the start of the study, data collectors tested the phone numbers provided by participants to investigate which phone lines were still active.

### Randomization

Upon confirmation of the operating phone numbers (n=411), we listed eligible women in random order using a random number generator. We selected every other woman, starting with the first woman, for the IFA arm (206/411, 50.1%); similarly, we selected every other woman, starting with the second woman, for the violence against women arm (205/411, 49.9%).

### Outcomes

The primary evaluation outcome for the mIFA arm is self-efficacy for IFA adherence. IFA use and anemia among women of reproductive age will also be measured as secondary outcomes. The primary evaluation outcome for the mBI arm is bystander self-efficacy in preventing violence against women.

### Data Collection

The mRANI study data collection will be embedded within the overall RANI trial, similar to its intervention delivery strategy. Women between the ages of 15 and 49 years residing in treatment (n=2061) and control (n=2049) clusters have been recruited and randomly selected to participate in the impact evaluation of the RANI project. Baseline survey of the RANI participants took place in September 2019, and an end line survey was scheduled to take place in February 2021. Baseline data collection for the larger RANI project served as formative data toward developing the mRANI EE programs and segmenting potential participants. The study team will conduct the impact evaluation for the mRANI study using data from end line assessments, controlling for baseline estimates.

The average time to complete a survey during the baseline and midline data collection was 55 minutes for all RANI measures and outcomes. The mRANI-specific modules take an average of 15 minutes to complete. Translation and back translation of the entire survey instrument, including mRANI-specific modules, will be conducted to check the accuracy of the questionnaire’s translation. Pretesting of mRANI-specific modules will be conducted in nonsampled villages to ascertain reliability and appropriateness in this intervention context. A description of the data collection methods for the parent trial can be found in the parent trial’s protocol [2].

### Overall Measures

#### Demographics

We will collect demographic information from the RANI sample, including age, education, number of children, and caste or tribe membership.

#### mIFA Measures

##### Self-efficacy for IFA Adherence

Self-efficacy in adhering to an IFA regimen was conceptualized as the extent to which women feel confident that they can continuously take IFA when facing a number of relevant barriers. At baseline, we asked the participants the extent to which they agreed they (1) could take IFA every week when not pregnant, (2) believed they could easily take IFA, (3) could take IFA even if their husband or father does not want them to, and (4) could take IFA even if their mother-in-law or mother does not want them to. Responses were coded on a 5-point Likert scale and averaged into a scale for self-efficacy for adherence at baseline.

We will measure self-efficacy for adherence at the end line using a modified scale that includes additional barriers for adherence. Participants will be asked the extent to which they are confident they (1) will continue to take IFA every day (for pregnant women) and every week (for nonpregnant women), (2) are anemic, (3) are not anemic, and (4) receive advice from others to discontinue taking IFA. Barriers were identified during the formative research phase of the RANI project [7]. Women will also be asked the extent to which they are confident they can take IFA every day if they are pregnant (or every week if they are not pregnant), as these are the recommended doses for this population. The four items will be answered on a 5-point Likert scale, ranging from *strongly disagree* to *strongly agree*. Scores will be averaged into a scale for self-efficacy to adhere to IFA at the end line.

#### IFA-Related Social Norms

The RANI project operationalizes three forms of social norms: descriptive, injunctive, and collective. This study conceptualizes IFA-related descriptive norms as the extent to which women believe others regularly consume IFA. At baseline, women were asked, “what proportion of a) non-pregnant, b) pregnant and c) adolescent girls take IFA regularly?” Responses were recorded as *none*, *some*, *about half*, *most*, or *all* and averaged into a three-item scale for descriptive norms related to IFA. Similar questions will be asked at the end line. Women will also be asked new descriptive norms questions at the end line related to adherence: “How many a) non-pregnant, b) pregnant c) adolescent girls do not miss a dose or two of IFA?” and “How many a) non-pregnant, b) pregnant and c) adolescent girls continue to take IFA even after missing a dose or two?”

IFA-related injunctive norms were conceptualized as the extent to which women believe others expect them to consume IFA regularly. At baseline, women were asked, “how many women in your community think you should take IFA tablets regularly if you are pregnant?” as well as if they are not pregnant. Responses were recorded on the same scale as descriptive norms, ranging from *none* to *all*. In addition, women were asked the extent to which they agreed that their (1) mothers-in-law (or mothers) and (2) husbands (or fathers) think they should take IFA regularly if they are pregnant, as well as not pregnant. Responses were recorded on a 5-point Likert scale ranging from *strongly disagree* to *strongly agree*. All responses were averaged into a 6-item scale for injunctive norms related to IFA use. Similar measures will be collected at end line. Similar to descriptive norms, women will also be asked new injunctive norms questions at the end line relating to adherence. We will ask women, “Do you agree that your a) mother-in-law (or mother) and b) husband (or father) will think badly of you if you miss a dose?”

Previous studies have conceptualized collective norms as the prevalence of a focal behavior within a community [47], and it has been operationalized as the mean of the behaviors of everyone in one’s community except that of the referent person, also called the *nonself mean* [48,49]. For the mIFA arm, we will operationalize collective norms as the prevalence of nonself mean of self-reported adherence to IFA supplements within a cluster, weighted by the number of women in the cluster who are also in the mRANI arm.

#### mBI Measures

##### Bystander Self-efficacy to Prevent Violence Against Women

This study conceptualizes bystander self-efficacy as the extent to which women feel confident in their ability to intervene when they witness another woman experiencing violence. At the end line, we will ask women the degree to which they agree with 8 statements stating, “If I witness violence against a woman, I am confident I could intervene by distracting (e.g. beating pots and pans); delaying (e.g. checking in after witnessing violence with the victim); delegating (e.g. seeking help from SHG group members or influential community leaders); documenting the violence (e.g. calling a hotline or recording on a cell phone) as a bystander.” Responses will be scored on a 5-point Likert scale, ranging from *strongly disagree* to *strongly agree*, and averaged into an eight-item measure for bystander self-efficacy to prevent violence against women.

##### Violence Against Women–Related Social Norms

Violence against women–related descriptive norms were conceptualized as the extent to which women believe that others intervene when witnessing violence in their community. We will measure descriptive norms by asking participants to report how many women in their community and friends would intervene if they saw a woman experiencing violence. Responses will be recorded as *none*, *some*, *about half*, *most*, or *all* and averaged across the two items.

Violence against women–related injunctive norms were conceptualized as the extent to which women believe that others expect them to intervene if they witness a woman experiencing violence. This study will operationalize these injunctive norms by asking participants the extent to which they agree with the following statement: “If you saw a woman experiencing violence, [referent] will expect you to intervene,” in which the referent will be (1) most women in the community, (2) most friends, and (3) most women in self-help groups. We will record responses on a 5-point Likert scale ranging from *strongly disagree* to *strongly agree* and average them across the two items.

The operationalization of violence against women–related collective norms will mirror the mIFA arm, except that we will use intentions to engage in bystander intervention strategies in lieu of behaviors [48,49]. As such, for the mBI arm, we will operationalize collective norms as the prevalence of nonself mean intentions to engage in bystander intervention strategies within a cluster, weighted by the number of participants in the cluster who are also in the mBI arm.

### Process Evaluation

Prior EE interventions leveraging IVR systems have reported success in their ability to facilitate and track audience engagement in real time and at scale [50]. Following their lead, monitoring of the mRANI intervention delivery and receptivity will occur in real time. Textbox 2 summarizes process evaluation measures and indicators for mRANI. Data on intervention dose, audience attention and involvement or engagement, character identification, narrative satisfaction and engagement, and perception of the most important problem in their village at the end of the EE program will be autogenerated by the IVR system immediately upon episode release and delivery. Process evaluation measures and indicators gauging recall and interpersonal communication related to mRANI will also be measured during the end line assessment.

**Mobile reduction in anemia through normative innovations process evaluation indicators.**

**Dose**

- Number of calls sent
- Number of calls received
- Number of entertainment education (EE) episodes heard in full by the mobile reduction in anemia through normative innovations (mRANI) participants

**Audience involvement or engagement**

- Number of interactions with interactive components
- Number of likes on episodes
- Number of replays of episodes
- Number of calls and queries on the mRANI hotline
- Number of referrals to community and clinical linkages

**Attention**

- Number of seconds spent listening to episodes
- Number of seconds spent relistening to episodes
- Number of seconds spent listening to jingles
- Number of seconds spent listening to episode teasers
- Number of seconds spent listening to episode recaps

**Recall**

- Percentage of participants who accurately recall prosocial themes addressed in the episodes
- Percentage of participants who accurately recall mRANI characters
- Percentage of participants who accurately recall major mRANI plot points

**Narrative satisfaction and engagement**

- Percentage of participants engaged with the narrative
- Percentage of participants satisfied with the narrative

**Character identification**

- Percentage of participants who identify with Malati (protagonist) or Dolly (protagonist’s daughter)

**Most important problem in the village**

- Percentage of participants who identify anemia or violence against women (depending on their assignment) as the most important problem at the end of the mRANI EE program

### Ethical Considerations

#### Research Ethics Approval

The full RANI trial, including the mHealth intervention, has been reviewed and approved by appropriate institutions in the United States and India. These institutions include the George Washington University institutional review board in the United States; the Sigma Science and Research in New Delhi, India; and the Indian Council for Medical Research’s Health Ministry’s Screening Committee. The trial has also been registered with the Clinical Trial Registry of India.

#### Participant Orientation, Consent, and Confidentiality

Participants will be contacted with preintervention delivery for validation of phone numbers, followed by orientation and enrollment. The orientation and enrollment activities will take place a week before the mRANI intervention delivery commences. A structured script will be prepared to orient potential participants to mRANI and inform them of the objectives and intervention delivery channel and duration. For participants who express interest in enrolling in the mRANI study, we will obtain verbal informed consent. Next, we will note their preferences for the day and time to receive the mRANI EE programs. Participants will also receive contact information for additional questions and feedback.

#### Risk Mitigation Plan

Given the short duration and digital delivery format of this intervention, we anticipate low levels of exposure to adverse events for mRANI participants. In addition, considering that the mRANI educational messages are strategically masked within an entertaining narrative tested in the field, we expect minimal sensitivity and negative reactions from our audience or their family members. However, the study team acknowledges that violence against women is a sensitive subject matter in Angul, Odisha. The mRANI study will adhere to the risk mitigation plan in place for the larger RANI trial [2]. Enrolled mRANI participant names and their data will be documented in an encrypted file. To maintain confidentiality, only 1 team member will have access to these data. To prevent unintended consequences for potential mRANI participants, the study team limited intervention delivery to women who are phone owners; thus, they have more agency and privacy related to their phone use. In addition, special consideration will be given to the delivery of EE programs during the day and time selected by the participant to ensure their privacy and security. The name of the mRANI study will appear on the caller ID when delivering new episodes so that participants and their family members will not face ambiguity tied to the source of the EE programs. Community and clinical referrals will be provided to participants who call the mRANI hotline. Local experts and consultants will triage requests beyond the scope and sphere of influence of the mRANI study. Finally, participants will have the option to opt out of mRANI EE programs at any time by contacting the mRANI hotline or by pressing 0 on their phone.

### Statistical Analysis

The primary analyses planned for the mRANI impact evaluation will test the hypotheses that participants assigned to the treatment arm will have higher levels of self-efficacy in IFA adherence (primary end point) and self-reported IFA use (secondary end point) at follow-up and that participants assigned to the control arm will have higher levels of bystander self-efficacy for prevention of violence against women. We will test these three hypotheses in three ways. First, because participants are randomized to treatment and control groups, a simple comparison of those groups should produce an unbiased estimate of the treatment effect. Therefore, we will estimate, for each end point, the linear regression model *Yij = b1 + b2 × TREATij + eij*, where *i* indexes the individual participant, *j* indexes the study cluster, and *b2* is the treatment effect. We will estimate this model by using ordinary least squares regression but use robust standard errors to test the null hypothesis (H_0_: b1=0) because of the nesting of participants in study clusters.

Although these analyses should produce unbiased estimates of treatment effects, they may miss key opportunities to increase statistical power and precision through two analytic elaborations. The first is to introduce cluster fixed effects to the model, which effectively results in a within-cluster comparison of the treatment and control group participants. As treatment assignment is within clusters, cluster and treatment assignments are orthogonal, so the addition of cluster fixed effects should increase statistical power and precision by reducing the residual error variance. In this case, the analytic model for each end point will be *Yij = aj + g1 × TREATij + eij*, where *aj* is a vector of cluster fixed effects, and *g1* is the treatment effect. As with the first modeling approach, we will estimate this model using ordinary least squares; however, in this case, the inclusion of cluster fixed effects obviates the need to use robust SEs.

Another opportunity to improve statistical power while retaining unbiased estimation is to control for pretreatment variables that are likely to be highly correlated with the primary and secondary end points. These include measures of the same variables taken before randomization: baseline and midline self-efficacy for serum hemoglobin levels, IFA adherence, and IFA use. Note that bystander self-efficacy for violence against women was not measured at baseline or midline and thus cannot be included as a covariate. In addition, sociodemographic variables assessed at baseline and midline, such as age, education, and parity, can also be considered for inclusion. We will choose covariates for inclusion based on their marginal associations with each end point. The resulting model for each end point will be *Yij = aj + d1 × TREATij + Xij × h + eij*, where *aj* is once again a vector of cluster fixed effects, *Xij* is a vector of select control variables assessed at baseline and midline, and *d1* is the treatment effect. As with the second modeling approach, we will estimate this model using ordinary least squares with no need for robust SEs.

This approach will produce three estimates of the treatment effect for each end point: b1, g1, and d1. All are unbiased, and we expect them to be similar across the three specifications. The main difference we expect would be in the SEs, which we expect will be highest in the first and lowest in the third specification. For transparency, we will report the estimates from all three specifications for each end point.

To assess the extent to which social norms and interactivity mediate the relationship between intervention exposure and primary outcomes in both mIFA and mBI arms, we will use a structural equation model to estimate the effects of the intervention on the mediator variables, the effects of the mediators on the 2 self-efficacy variables (IFA use and bystander intervention), and the direct effect of the treatment arm on both self-efficacy variables. From this model, we will estimate the percentage of the intervention effect on each aspect of self-efficacy mediated by each of the mediators [51]. All analyses will be performed using Stata (StataCorp) for Windows, version 14.2 [52].

## Results

Data collection for the mRANI intervention is integrated within the parent RANI trial. Household surveys collected responses for the primary and secondary outcomes of the mRANI study between February and March 2021. End line data were collected from all 381 (192/381, 50.4% women in the IFA treatment arm and 189/381, 49.6% women in the violence against women’s control arm) study participants. Data analysis is expected to be completed by October 2021.

## Discussion

### Principal Findings

The primary aims of the mRANI intervention are three-fold. First, we wish to determine whether an intervention delivered through a rather thin medium, an IVR channel, can propel behavior change. By relying on a social norms–based theory [21,47,53] as the underlying conceptual guide and an EE theme to capture listeners’ attention, we believe we can improve participants’ self-efficacy. In resource-constrained settings, where internet access is low, voice calls may be one of the most effective ways to reach women [54-56]. As shown by our mHealth user data, most women in these communities own basic phones, and voice messages may be an efficient way to reach them. This may be particularly true in these communities and other rural areas in India, as our formative research showed that women do all the household work and also often work outside the home to earn money. Therefore, their time is limited, and brief voice calls may be more convenient than in-person meetings for social behavior change interventions.

The second aim of the study is to determine whether embedding an mHealth trial within the intervention arm of a larger field trial can further add to the effects of the parent trial. We will use these data to inform the next iteration of the RANI intervention. Finally, we also wish to determine whether the reciprocal control double intervention design can yield meaningful results.

This study does have a few noteworthy limitations. First, the generalizability of our study is confined to women who are cell phone owners. Although ownership is increasing rapidly in India [28], only 43.97% of women in our sample are cell phone owners. It may be that the factors limiting ownership (including low socioeconomic status, being a member of a marginalized group, and lack of power in interpersonal relationships) also drive poor health. Study generalizability is also compromised as those participating in the parent RANI trial (who form the basis of our recruitment) may not be representative of the larger population. Despite these limitations, we envision robust internal validity, given the randomized nature and longitudinal components of the study design.

### Dissemination

In spring 2021, at the end of the RANI evaluation, we will hold a virtual convening in Bhubaneswar, India (the capital of Odisha), to present the mRANI findings along with the main trial findings. We will invite key stakeholders from Angul, such as district officials, program planners, researchers, and policy makers working on anemia reduction efforts in Odisha. We will also disseminate findings locally back to the communities where the intervention will take place through smaller presentations and dissemination materials.

### Conclusions

To our knowledge, an mHealth intervention has not been used to reduce anemia among nonpregnant women, the subpopulation that makes up the largest number of women with anemia [1,29]. As anemia continues to affect women’s work productivity, overall health, and the health of future generations, it is critical to explore innovative ways to increase IFA use and reduce anemia levels. If mRANI proves to be effective, other districts and states within India and Southeast Asia may follow suit, thus reducing the overall global burden of anemia among women.

Finally, although the inception of this mRANI intervention predated the COVID-19 pandemic, the need to reach women via mobile phones rather than in-person settings is critical now more than ever. Given the uncertainty of when and how in-person interventions will unfold, contactless interventions are critical to be able to continue providing information to communities staying home. Furthermore, violence against women was rampant before the COVID-19 pandemic; however, research shows that the incidence is rising worldwide and specifically in India [57,58]. mRANI may provide an efficient, cost-effective medium to reduce this incidence and change social norms around bystander intervention against violence against women.

## Appendix

Multimedia Appendix 1 Extended details on story testing.

Multimedia Appendix 2 Overall mobile reduction in anemia through normative innovations entertainment education narrative.

Multimedia Appendix 3 Episode summaries for both mobile reduction in anemia through normative innovations arms.

## Abbreviations

EE: entertainment education
IFA: iron folic acid
IVR: interactive voice response
mBI: mobile bystander intervention
mHealth: mobile health
mIFA: mobile iron and folic adherence
mRANI: mobile reduction in anemia through normative innovations
RANI: reduction in anemia through normative innovations
RCDT: reciprocal control double treatment
