# Supplementary material for: mHealth for Anemia Reduction: Protocol for an Entertainment Education–Based Dual Intervention
Source: JMIR Res Protoc. 2021 Nov 22;10(11):e26252. doi: 10.2196/26252 (PMC8663628; doi:10.2196/26252)
Supplement: Multimedia Appendix 1 [file resprot_v10i11e26252_app1.docx]

**Extended details on mRANI Story testing**

The entertainment education storyline was developed through an iterative process. The study team decided early on to focus initially on the entertainment aspect of the story, developing a story arc with protagonists, antagonists, multidimensional characters, and an aspirational ending. The aim was to develop a broad storyline within which we could incorporate sub-plots of story arcs that address the educational and theoretical aspects of the control and treatment arms (i.e. self-efficacy for IFA adherence and bystander intervention for VAW respectively). We started off with four storylines followed by a meaningful deliberation within the study team on the merits of all four plots. The team decided to move forward with two contextually suitable and entertaining plots for field testing in order to select one that would be most liked and accepted by our intended audience.

Two stages of field testing were conducted in a similar rural setting as the treatment site for the larger RANI intervention. The first stage of field testing involved reading the two stories out loud to five female participants between the ages of 15 to 35 years old. A qualitative topic guide was developed to test the two storylines. Following the narration of the two stories, we collected participant responses using the qualitative topic guide which included semi-structured questions on what they liked or did not like about the story, whether and why they would share the story with women in their villages, if they could identify with the protagonist, whether the narrative moved them emotionally, and finally, how they perceived other women in their communities would react to the story. We also asked our participants to rank the two stories in their order of preference and state their rationale for their ranking. Informed with results from the first stage of story testing, the team selected a storyline to test in the second stage of story testing. The finalized storyline was audio-recorded and played for three additional participants in the same age range. In addition to seeking feedback on topics listed earlier, the audio-recording of the story was paused midway to gauge their interest in listening to the remainder of the story. Results from both rounds of story testing were utilized to modify the finalize the overall storyline within which the educational and theoretical sub-plots would then get incorporated.

**This is a Multimedia Appendix to a full manuscript published in the J Med Internet Res. For full copyright and citation information see** [**https://www.researchprotocols.org/2021/11/e26252**](https://www.researchprotocols.org/2021/11/e26252)**.**
