# Supplementary material for: mHealth for Anemia Reduction: Protocol for an Entertainment Education–Based Dual Intervention
Source: JMIR Res Protoc. 2021 Nov 22;10(11):e26252. doi: 10.2196/26252 (PMC8663628; doi:10.2196/26252)
Supplement: Multimedia Appendix 2 [file resprot_v10i11e26252_app2.docx]

**Overall Narrative**

Malati is a content housewife who lives in a rural village in India with her teenage daughter Dolly who sometimes aspires to be more than just a housewife. Her husband, Narayan, works as a migrant bookkeeper in Surat. The family has been facing financial distress due to the unabated spread of COVID-19 in India. They reach a crisis point when Narayan is laid off from his work. He attempts to find other positions but is left with no choice but to return to his village. When Narayan finally makes it home safe and sound after the government mandated quarantine, the family is surprised with and burdened by expenses beyond their current means when Dolly is unexpectedly diagnosed with an illness. Malati is ultimately left with no option other than to mortgage her ancestral jewellery and home for a loan to tide over the difficult situation. These series of unfortunate events eventually lead Malati towards empowerment, support from a community of women in her village, financial independence, and better health and livelihood for herself and her family.

**This is a Multimedia Appendix to a full manuscript published in the J Med Internet Res. For full copyright and citation information see** [**https://www.researchprotocols.org/2021/11/e26252.**](https://www.researchprotocols.org/2021/11/e26252)
