# Supplementary material for: mHealth for Anemia Reduction: Protocol for an Entertainment Education–Based Dual Intervention
Source: JMIR Res Protoc. 2021 Nov 22;10(11):e26252. doi: 10.2196/26252 (PMC8663628; doi:10.2196/26252)
Supplement: Multimedia Appendix 3 [file resprot_v10i11e26252_app3.docx]

**Episode Summaries for the two mRANI arms**

Table A1 Overlap and distinction between the two narratives

| **IFA arm** | **Bystander arm** |
| --- | --- |
| The virus is spreading and Narayan, Malati and Dolly are worried about finances and Narayan’s safety while he works in another city. | The virus is spreading and Narayan, Malati and Dolly are worried about finances and Narayan’s safety while he works in another city but they get distracted and distressed by the routine physical violence between their elderly neighbors. Tensions begin flaring between Narayan and Malati. |
| Narayan returns to the village only to find out he has to quarantine upon his return before he gets to go home to his family. | Narayan heads to the bus station only to find out he has to quarantine upon his return before he gets to go home to his family. He witnesses an eve teasing incident. A group of young women record and upload the video on social media which goes viral. Tensions continue to build between Narayan and Malati. Dolly urges her mother to become more assertive and set healthier boundaries with her father. |
| While Narayan is in quarantine Malati and Dolly worry about the possibility of the virus infecting him while they slowly run out of savings. | While Narayan is in quarantine Malati and Dolly worry about the possibility of the virus infecting him while they slowly run out of savings. Malati visits Sukuti and Sukuti shows her a viral social media video from the bus station. In response, Malati shares how they witness her elderly neighbor’s domestic violence but feels disempowered to do anything. Sukuti mentions how she’s been learning about the 4Ds of bystander intervention at her SHG then Malati and her discuss whether it’s high time for social tolerance when it comes to VAW. |
| Narayan finally returns home from quarantine safe and healthy but Dolly is now symptoms of infection. | Narayan finally returns home from quarantine safe and healthy but Dolly is now showing symptoms of infection. Tensions increase between Malati and Narayan as his financially controlling behaviors intensify under the stress of quarantine, unemployment, and the pandemic. |
| Narayan and Malati take Dolly to the hospital where they discover Dolly has the flu and anemia. They need funds fast to manage household and hospital expenses. | Narayan and Malati take Dolly to the hospital where they discover Dolly has a reproductive health infection. They need funds fast to manage household and hospital expenses. Malati witnesses’ workplace verbal abuse directed at Dr. Patel by a senior health care professional to which a bystander (fellow female physician) intervenes by distracting the abuser and supporting Dr. Patel if she chooses to report the behavior. Malati begins to internalize the message that social change is necessary so all women no longer face routine violence throughout their lives. |
| Malati has no choice but to borrow money from a moneylender until Narayan finds work in the village. Will Narayan agree with her decision? | Malati has no choice but to borrow money from a sahukar until Narayan finds work in the village. At the sahukar’s she hears his daughter in law being physically abused. She speaks up until the Sahukar intervenes and asks his wife to distract their son. When she returns home, Narayan is outraged that she’d take such a step. Tensions are now at the boiling point for Narayan and Malati. Malati is now determined to become financially independent. |
| Narayan and Malati question why fate is putting them such through such hardships after realizing Malati may have anemia as well. Malati visits her friend Sukuti for consolation and guidance. | Narayan and Malati question why fate is putting them such through such hardships after discovering Dolly’s reproductive infection. Malati visits her friend Sukuti for consolation and guidance. They continue their discussion on how VAW is a lived reality for women of all ages and social class in society. Sukuti shares that many self-help groups in neighboring districts are beginning to say Enough is Enough and they’re trying to do something. Malati wonders if they should be doing something similar in their own village. Sukuti convinces Malati to visit the SHG group to help her resolve her health and financial issues. |
| Sukuti and Malati visit the village SHG group after Sukuti insists Malati go with her. Malati discovers a whole new world of opportunity | Sukuti and Malati visit the village SHG group after Sukuti insists Malati go with her. Malati discovers a whole new world of opportunity |
| Sukuti and Malati visit the village SHG group after Sukuti insists Malati go with her. Malati discovers a whole new world of opportunity | Malati visits Tapa with Sukuti and SHG leader to learn about a new business opportunity. To start the business, she needs to come up with half the funds. Already in debt, Malati wonders if she can manage borrowing more money to run a business successfully without risking the family’s future. Malati, Tapa and her husband bear witness to verbal abuse directed towards Tapa’s client. Her husband Gopal immediately intervenes by distracting the harasser. |
| Malati and Narayan decide to bet on their health which they believe will lead to the way to wealth. Malati commits to becoming anemia mukt (free) and begins taking iron supplements regularly with influenced by the unwavering support and expectations coming from Sukuti and her SHG sisters’. | Malati and Narayan are now at the tipping point of their tensions when Malati suggests mortgaging their home. Malati gets through to her Narayan and explains why her success is the family’s success. Malati and Narayan decide to bet on their health which they believe will lead to the way to wealth. |
| Malati has set up her business and it is taking off. Dolly and Sukuti help her out. She is slowly becoming healthier and pushing Dolly to make commit to taking iron batika regularly with Sukuti’s help. Narayan continues to look for work in the village. The family hopes and prays he won’t have to go work far from them in another city. | Malati has set up her business and it is taking off. Dolly and Sukuti help her out. The group (Malati, Dolly, Sukuti) bears witness to the elderly neighbor’s domestic violence again but this time they decide to intervene as a collective by starting a movement to live stream them beating pots and pans continuously until the day arrives that a woman hasn’t experienced violence in the village. Narayan continues to look for work in the village but wants Malati to focus on the business instead of her activism. Dolly intervenes and tells Narayan he can longer control her mother or treat her poorly. |
| Things are finally starting to turn around for Malati, Dolly and Narayan. They have paid off their loans. Narayan faces another job rejection and Dolly is convinced she will do poorly in her college entrance exam having run out of time she felt she needed. | Things are finally starting to turn around for Malati, Dolly and Narayan. They have paid off their loans. Narayan faces another job rejection and Dolly is convinced she will do poorly in her college entrance exam having run out of time she felt she needed. |
| The family discovers Malati was offered a college scholarship due to ranking the highest in the district in her entrance exams combined with her matric exams. Narayan receives a job offer from Malati’s newly established Dairy cooperative. Journalists come to cover the family’s rise from poor health and financial issues to financial abundance and becoming anemia free. | Malati receives media recognition for her viral activism and becomes a highly followed social influencer. A journalist comes to cover Malati and her family. Malati drives home the idea that everyone has a role to play in becoming good upstanders and curbing violence against women in all forms. |

**This is a Multimedia Appendix to a full manuscript published in the J Med Internet Res. For full copyright and citation information see** [**https://www.researchprotocols.org/2021/11/e26252.**](https://www.researchprotocols.org/2021/11/e26252)
